# Ordinal Deep Learning for Lumbar Foraminal Stenosis Grading on Sagittal MRI

**DOI:** 10.3390/jimaging12080388

**Published:** 2026-08-19

**Authors:** Rohan A. Phadke, Samer G. Salman, Zane G. Salman, Akhil Marupudi, Kirtan Patel, Joshua Ong, Alireza Tavakkoli, Sainyam Galhotra, Ajay Tripuraneni, James Rizkalla, Nathan J. Lee

**Affiliations:** 1 School of Medicine, Baylor College of Medicine, Houston, TX 77030, USA; samer.salman@bcm.edu (S.G.S.);; 2 College of Natural Sciences, The University of Texas at Austin, Austin, TX 78712, USA; 3 McGovern Medical School, The University of Texas Health Science Center, Houston, TX 77030, USA; 4 Edward Via College of Osteopathic Medicine-Louisiana, Monroe, LA 71203, USA; 5 Massachusetts Eye and Ear, Harvard Medical School, Boston, MA 02114, USA; 6 Department of Computer Science, University of Nevada, Reno, NV 89557, USA; 7 Department of Computer Science, Cornell University, Ithaca, NY 14853, USA; 8 Department of Orthopaedic Surgery, Baylor University Medical Center, Dallas, TX 75246, USA; 9 Midwest Orthopaedics at Rush, Rush University School of Medicine, Chicago, IL 60612, USA

**Keywords:** lumbar foraminal stenosis, spine MRI, ordinal classification, interpretable machine learning, morphometry, image segmentation, deep learning, spinal decompression

## Abstract

Lumbar foraminal stenosis grading contributes to surgical-level selection, but automated four-grade classification remains challenging. Published pipelines for this dataset reach approximately 65% four-class accuracy, and deep classifiers offer no anatomical rationale. We investigated whether interpretable, millimeter-scale morphometry from segmentation masks improves grading beyond a deep image model. We analyzed the LSS-MRI-AISSLab sagittal T2-weighted dataset (469 patients, 2979 expert-graded foramina spanning L1-L2 through L5-S1 bilaterally on a four-grade scale). Ten morphometric descriptors were computed from mid-sagittal polygon segmentations and scaled to millimeters using each patient’s recorded pixel spacing. A dual-branch network combined a fine-tuned ResNet-18 embedding of each foraminal region of interest with the morphometric vector through an ordinal regression head. Foraminal regions were supplied from expert bounding-box annotations; automated localization within the full sagittal examination was not evaluated. Four configurations (nominal softmax, appearance-only, anatomy-only, and fusion) were compared on a locked patient-level test set of 94 patients after five-fold cross-validation, with quadratic weighted kappa (QWK) as the primary endpoint and patient-clustered bootstrap inference. Feature-grade correlations were reported pooled and adjusted for lumbar level. Fusion achieved QWK 0.813 (95% confidence interval [CI] 0.769–0.847) and 75.3% four-class accuracy. Appearance-only was statistically indistinguishable (QWK 0.806; delta QWK +0.006, 95% CI −0.023 to 0.036, *p* = 0.68), whereas anatomy-only reached 0.444, and a level-and-side-only reference reached 0.314. Boundary discrimination was strong (area under the curve 0.92–0.99), 98.3% of predictions fell within one grade, and performance was consistent across scanner vendors. Morphometric associations were confounded by level: the apparent spondylolisthesis effect (rho −0.349) disappeared after adjustment (rho −0.000), while disc height, null when pooled (rho +0.021), emerged as a genuine within-level effect (rho −0.108). A fine-tuned ordinal image classifier achieved strong agreement for four-grade lumbar foraminal stenosis classification. The evaluated segmentation-derived morphometric features did not improve performance beyond imaging alone, and several apparent anatomic associations reflected confounding by lumbar level. External and prospective validation in complete clinical MRI workflows are needed before implementation.

## 1. Introduction

Lumbar spinal stenosis is among the most prevalent causes of back and leg pain in the aging population, accounting for approximately 600,000 US surgical procedures annually [1]. Surgical benefit over nonoperative care is durable but attenuates over long-term randomized and cohort follow-up [2,3,4,5], and stenosis is not inevitably progressive without surgery [6]. Foraminal stenosis carries particular weight in surgical planning: moderate and severe foraminal narrowing on MRI independently predicts eventual surgery (odds ratios 2.22 and 2.12 over long-term follow-up) [7], and severe preoperative foraminal stenosis predicts a lower probability of meaningful improvement after posterior decompression (odds ratio 0.22 in the NORDSTEN trial) [8]. Yet degenerative narrowing is also common on MRI in asymptomatic adults, and radiographic severity correlates only weakly with symptoms and disability [9,10,11]. Imaging severity therefore informs rather than determines operative decision-making, which also weighs symptoms, examination and electrodiagnostic findings, the response to selective nerve root injection, coexisting central canal or lateral recess disease, and segmental instability. Operative strategies for degenerative lumbar disease span open and endoscopic decompression, pars repair, and motion-preserving arthroplasty [12,13,14].

The Lee grading system, a four-grade ordinal scale based on progressive perineural fat obliteration and nerve root morphologic change on sagittal MRI, is a widely adopted foraminal stenosis classification [15]. For the central canal, the Schizas morphologic grades and the Lee central-canal system play an analogous role [16,17,18]. The foraminal system’s original validation reported nearly perfect inter- and intra-observer agreement (kappa 0.80–1.0) between two radiologists, but subsequent multi-reader studies find substantially lower reliability, with Fleiss’ kappa as low as 0.295–0.311 across mixed-experience cohorts [15,19]. This variability, concentrated in the intermediate grades, motivates standardization efforts, including consensus radiologic criteria [20,21]; reader education [22]; and, as here, automated grading [19].

Deep learning has shown promise for lumbar stenosis classification on MRI: a meta-analysis of 48 studies reported pooled accuracy of 0.885 for binary stenosis detection, but polytomous grading accuracy fell to 0.503 for mild and 0.512 for moderate disease [23]. For neural foraminal stenosis specifically, a two-stage convolutional network reached kappa 0.89 for dichotomous grading, near the 0.94–0.95 of two subspecialist radiologists [24], and a three-stage network reached AUROC 0.95 for presence-versus-absence classification, exceeding the radiologist average of 0.88 [25]. Backbone choice also materially affects multi-class performance in related musculoskeletal imaging tasks [26]. Most high-performing systems, however, dichotomize severity (normal/mild versus moderate/severe) rather than preserving the four-grade ordinal resolution surgeons use [24,27]. Whether interpretable, segmentation-derived morphometric features improve four-class ordinal grading beyond deep image embeddings, and whether they add value beyond lumbar level alone, remains unanswered.

## 2. Methods

### 2.1. Dataset and Cohort

We analyzed sagittal T2-weighted lumbar magnetic resonance imaging (MRI) from the publicly available LSS-MRI-AISSLab dataset, in which 469 of 500 patients carry expert foraminal annotations: 2979 graded neural foramina from L1-L2 through L5-S1 bilaterally, each labeled on a four-grade ordinal scale (0, normal; 1, mild; 2, moderate; 3, severe) with laterality and disc level recorded separately. Imaging spanned multiple vendors and protocols (in-plane matrices 256 by 256 to 1008 by 1008; pixel spacing 0.396 to 1.25 mm); every annotation is stored at native resolution, so physical measurements multiply pixel distances by that patient’s Digital Imaging and Communications in Medicine (DICOM) pixel spacing, without rescaling. One patient’s single labeled foramen was annotated on a slice with no corresponding image in the dataset’s detection image set; with no region of interest (ROI) extractable, that patient and foramen were excluded (leaving 468 patients and 2978 foramina); the exclusion fell in the development split, so the locked test set was unaffected.

### 2.2. Morphometric Feature Extraction

Ten interpretable descriptors were computed per disc level from the mid-sagittal polygon segmentations, all in millimeters: disc height and its ratio to mean adjacent vertebral body height; upper and lower vertebral body heights; spondylolisthesis offset, the anteroposterior (AP) displacement between adjacent vertebral body centroids, and its ratio to vertebral body depth; segmental angulation; disc AP width; and the distances from the disc centroid to the two annotated posterior element structures. Coming from a single midline slice, the features are level-specific, not side-specific: left and right foramina at one level receive identical morphometry, separated only by the side indicator. Mid-sagittal features were used because only that slice carries expert polygon segmentations; parasagittal slices exist in the dataset (the foraminal bounding boxes themselves lie on parasagittal images) but are not segmented, and midline disc-vertebral geometry was expected to track foraminal narrowing mechanically, through disc collapse and vertebral slip. At L5-S1 the caudal structure is the sacrum, so centroid-offset and angulation features were treated as missing; these were absent for 315 foramina (10.6%; 278 at L5-S1, 37 from incomplete segmentations), and all ten descriptors were absent for 44 (1.5%). Missing values were imputed with the training-fold median and standardized with training-fold statistics only, so no information crossed the split. The anatomy branch also receives level indicators, so the network can learn level-conditional offsets around a global imputation constant; sensitivity analyses nonetheless retrained the anatomy-only model with level-stratified median imputation (global fallback at L5-S1, where centroid features are undefined) and k-nearest-neighbor imputation (k = 5).

### 2.3. Network Architecture and Training

Each foramen was cropped from its bounding box with a 40% peri-foraminal margin and passed to an ImageNet-pretrained ResNet-18 convolutional neural network (CNN). The morphometric vector, concatenated with level and side indicators, was encoded by a small multilayer perceptron; the joined representations were read out by a conditional ordinal regression for neural networks (CORN) head, which predicts the probability of exceeding each successive severity threshold so that a normal-to-severe error costs more than a normal-to-mild one. Four configurations were trained (nominal softmax, which ignores ordering; appearance-only; anatomy-only; and fusion) using AdamW with separate backbone and head learning rates, grade-balanced sampling, geometric and intensity augmentation, and early stopping on validation agreement.

### 2.4. Statistical Analysis

Patients, not foramina, were the unit of splitting because one spine contributes many foramina. Twenty percent of patients formed a locked test set; the remainder entered five-fold cross-validation stratified by each patient’s highest grade, and test predictions were averaged across fold models. The rule converting probabilities to a grade was selected out of fold from three candidates, taking the simplest within 0.01 kappa of the best (arg max for all models except a threshold rule for anatomy-only); the locked test labels never influenced the choice. The primary endpoint, quadratic weighted kappa (QWK), rewards near misses and penalizes distant errors. Secondary endpoints were four-class accuracy, balanced accuracy, adjacent accuracy (within one grade), per-grade sensitivity and specificity, area under the receiver operating characteristic curve (AUROC) at each ordinal boundary, and expected calibration error (ECE). Confidence intervals (CIs) come from a patient-clustered bootstrap (2000 resamples of patients; 5000 for the fusion-minus-appearance difference). Because segment geometry and stenosis prevalence both vary down the lumbar spine, every feature-grade Spearman correlation is reported pooled across levels but adjusted for lumbar level.. A reference model using only level and side indicators, fitted on development data, quantified how much of the anatomy branch reflects level alone. The full analysis pipeline and the exact patient-level train/test split indices are openly available at https://github.com/rohanpa27/LSS_foraminal_stenosis_imaging (accessed on 12 August 2026) under an open-source MIT license.

## 3. Results

### 3.1. Cohort and Morphometric Associations

After ROI extraction, 2978 foramina from 468 patients entered modeling: 374 patients with 2398 foramina in the development set, and a locked test set of 94 patients with 580 foramina (378 normal, 104 mild, 53 moderate, and 45 severe), preserving the level-dependent imbalance of the source data. Pooled morphometric correlations proved misleading because level drives both geometry and disease (Supplementary Table S1, Supplementary Figure S1). The centroid offset defining spondylolisthesis shifted monotonically down the lordotic spine (+6.9 mm at L1-L2 to −5.1 mm at L4-L5), while the share of normal foramina fell from 94% to 39%; consequently, its strong pooled correlation (rho −0.349) vanished after level adjustment (rho −0.000, 95% CI −0.055 to 0.053), as did segmental angulation (+0.211 to −0.036) and Posterior A distance (+0.146 to −0.002). The reverse also held: disc height, null pooled (rho +0.021), was consistently negative within level (−0.108, 95% CI −0.163 to −0.054; negative at all five levels), matching the surgical expectation that a collapsing disc narrows its foramen. Only disc AP width kept most of its signal (+0.296 to +0.195).

### 3.2. Grading Performance

**Figure 1:**
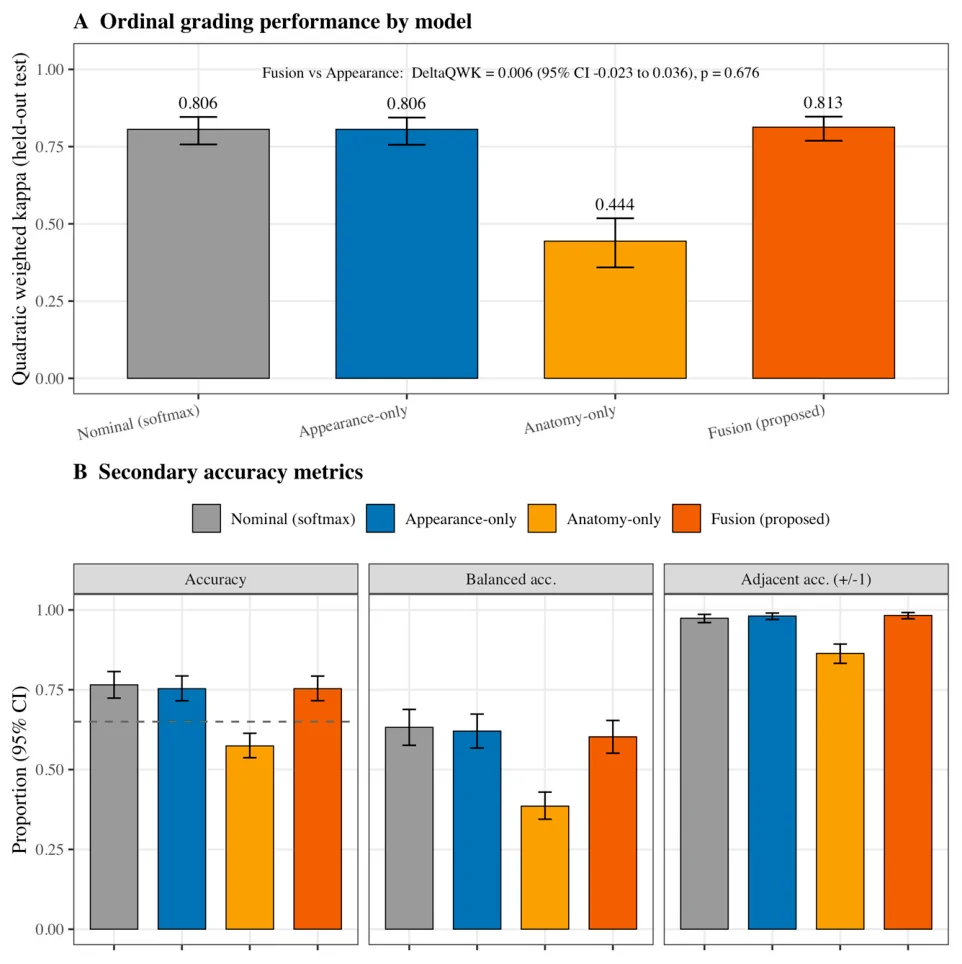
Ordinal grading performance across model configurations (held-out test). Error bars are patient-clustered bootstrap 95% confidence intervals (2000 resamples of patients). (**A**) QWK; the fusion and appearance-only models were statistically indistinguishable (delta QWK + 0.006, *p* = 0.676). (**B**) Secondary accuracy metrics; dashed line marks the approximately 65% published benchmark four-class accuracy.

On the locked test set, the fusion model reached QWK 0.813 (95% CI 0.769–0.847) with 75.3% four-class accuracy, above the approximately 65% previously reported for this dataset (Table 1, Figure 1A). Appearance-only was statistically indistinguishable from fusion (QWK 0.806, 95% CI 0.756–0.844; delta QWK +0.006, 95% CI −0.023 to 0.036, *p* = 0.68), and nominal softmax matched it (0.806). Anatomy-only reached 0.444 (95% CI 0.359–0.518), but a reference model given only level and side reached 0.314 (95% CI 0.266–0.360), so level and side alone reproduce roughly seven tenths of the anatomy branch’s agreement (QWK ratio 0.314/0.444 = 0.71; Table 1). Secondary metrics followed the same pattern with overlapping intervals (Figure 1B), and adjacent accuracy was 98.3% for fusion. Fusion’s higher QWK despite lower balanced accuracy and macro F1 than nominal softmax (Table 1) reflects the metrics themselves: ordinal training trades exact hits on scarce intermediate grades for fewer large-magnitude errors, which QWK rewards but class-averaged metrics do not. Imputation choice did not explain the anatomy branch’s weakness: level-stratified median and k-nearest-neighbor imputation gave test QWK 0.449 and 0.412, versus 0.463 for a matched rerun with the global median (differences −0.014 and −0.051; 95% CIs −0.068 to 0.037 and −0.101 to −0.004).

### 3.3. Discrimination, Calibration, and Error Structure

**Figure 2:**
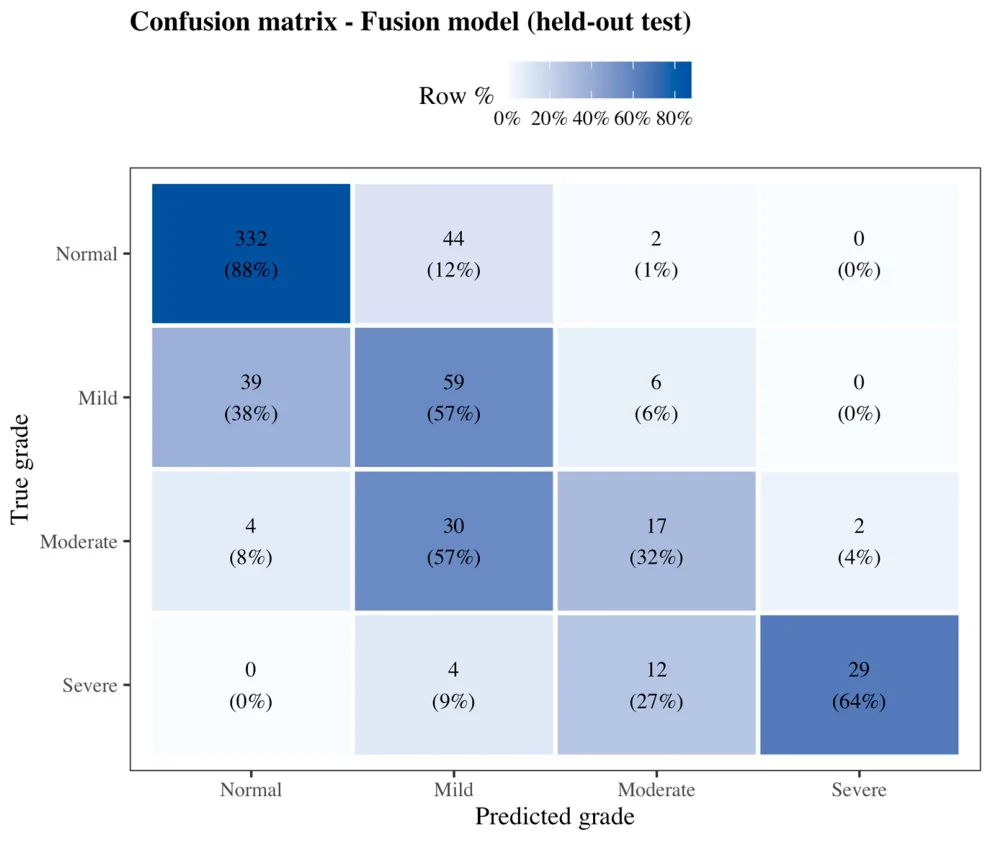
Row-normalized confusion matrix for the fusion model on the held-out test set (cell counts and row percentages shown).

**Figure 3:**
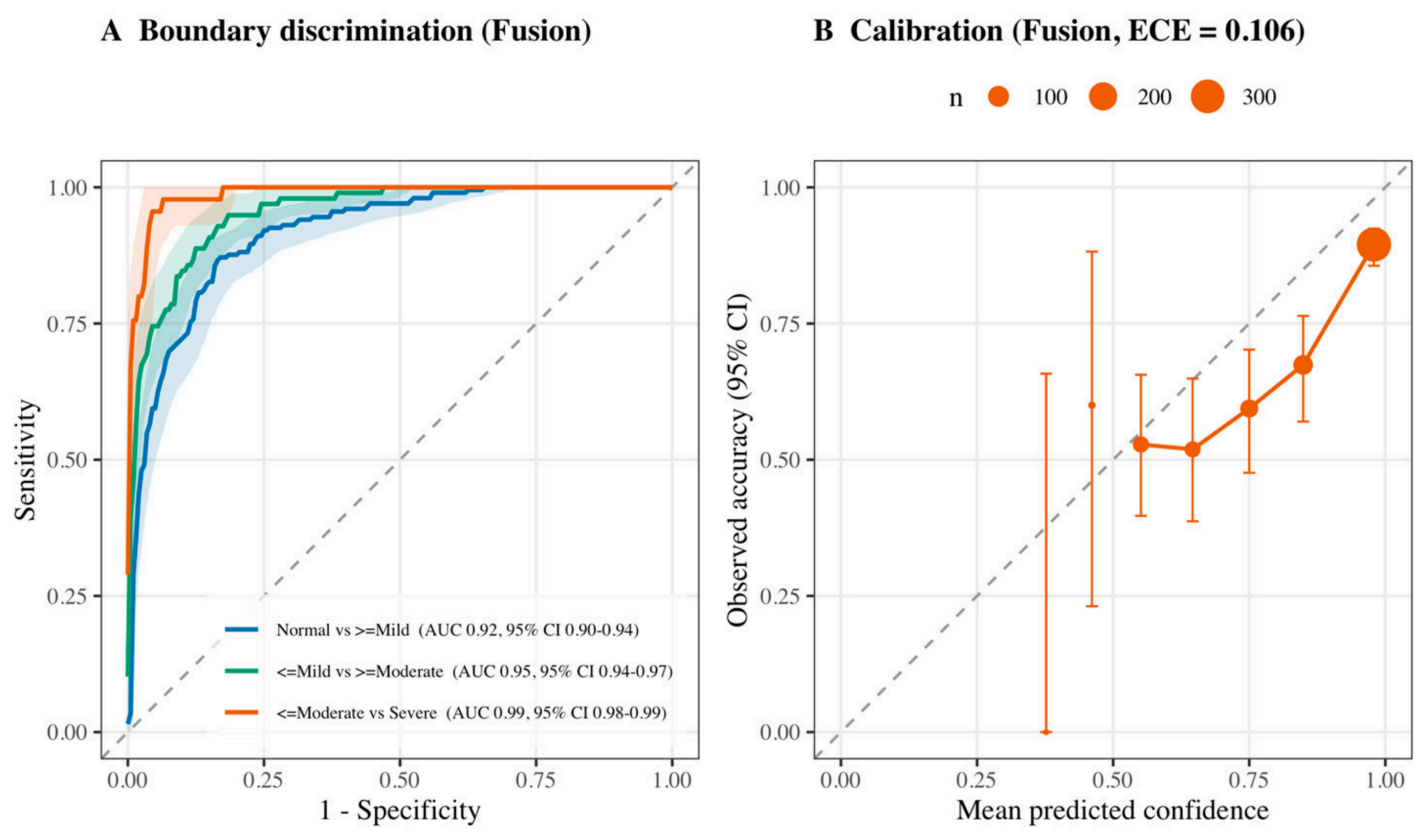
(**A**) Receiver-operating characteristic curves at the three ordinal boundaries for the fusion model; shaded bands are patient-clustered bootstrap 95% confidence intervals and legend entries give AUROC with its interval. (**B**) Reliability diagram; error bars are Wilson 95% intervals on observed accuracy, point area is proportional to the number of predictions in the bin, and the connecting line spans only bins holding at least 10 predictions. Expected calibration error 0.106.

Per-grade behavior was strongly asymmetric (Table 2a). Sensitivity was high for normal foramina (0.878, 95% CI 0.844–0.912) but fell to 0.321 (95% CI 0.203–0.444) for moderate stenosis; specificity rose with severity to 0.996 for severe disease. Errors concentrated on the mild-to-moderate boundary, with essentially no normal-to-severe misclassification (Figure 2). Discrimination at the ordinal boundaries was nonetheless strong (Table 2b, Figure 3A): AUROC 0.917 (95% CI 0.895–0.939) for normal versus any stenosis, 0.953 for moderate or worse, and 0.987 for severe. Calibration was moderate (ECE 0.106): over-confident through the middle of the confidence range, well calibrated only at high confidence (Figure 3B). Post-hoc recalibration (temperature or Platt scaling) was not evaluated; such monotone transformations leave discrimination and grade assignment unchanged, but recalibration should precede clinical use of the confidence outputs.

### 3.4. Performance by Level and Scanner

**Figure 4:**
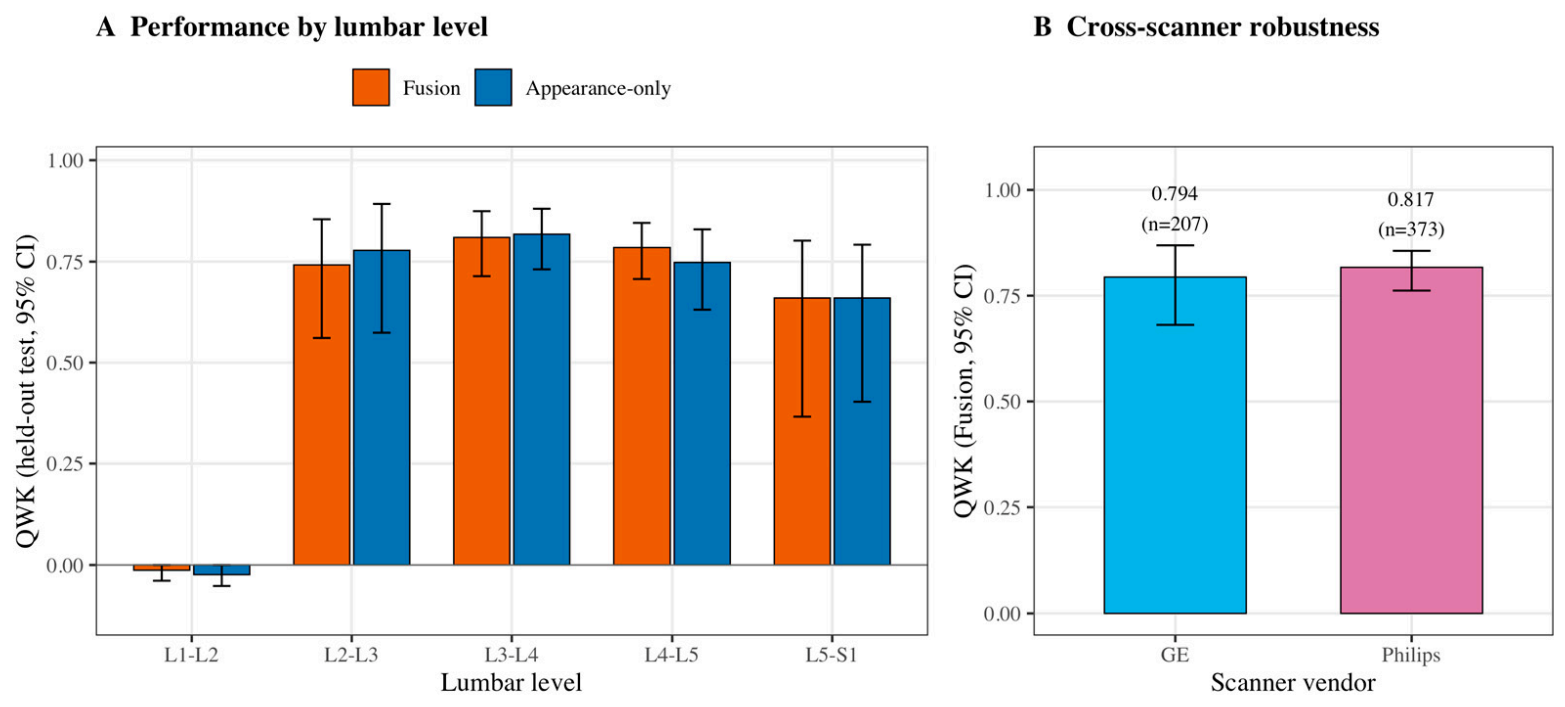
(**A**) QWK by lumbar level, fusion versus appearance-only. (**B**) Cross-scanner robustness of the fusion model by MRI vendor. Error bars are patient-clustered bootstrap 95% confidence intervals; intervals overlap at every level, and L1-L2 carries almost no higher-grade disease so its kappa is uninformative.

Agreement varied by lumbar level (Supplementary Table S2a, Figure 4A). It was highest at L3-L4 (QWK 0.810) and L4-L5 (0.785), the most annotated and balanced levels; lower at L5-S1 (0.660); and uninformative at L1-L2 (−0.013, 95% CI −0.039 to 0.000), where higher-grade disease is essentially absent (test set: 107 normal, 6 mild, 1 moderate, and 0 severe; the full dataset holds no severe L1-L2 foramen). Fusion and appearance-only intervals overlapped at every level. Performance was consistent across scanner manufacturers (Supplementary Table S2b, Figure 4B): QWK 0.794 (95% CI 0.681–0.869) on GE and 0.817 (95% CI 0.762–0.856) on Philips systems, indicating the model did not simply learn vendor-specific appearance.

## 4. Discussion

This study evaluated a dual-branch architecture fusing a fine-tuned ResNet-18 image embedding with segmentation-derived morphometric features for four-grade ordinal grading of lumbar foraminal stenosis on sagittal T2-weighted MRI. The fusion model achieved QWK 0.813 and 75.3% four-class accuracy, exceeding the approximately 65% previously reported on this dataset, yet the central finding was negative: the morphometric branch contributed no statistically significant improvement over the appearance-only model (delta QWK + 0.006, 95% CI − 0.023 to 0.036, *p* = 0.68), and most of its apparent univariate signal reflected lumbar level confounding rather than segment-specific pathology.

This performance aligns with the broader trajectory of deep learning for spinal stenosis grading, part of a wider machine-learning effort across spine care [28], in which binary tasks succeed (pooled accuracy 0.893 for neural foraminal stenosis) but polytomous grading falls sharply for intermediate grades [23]. The model’s strong boundary discrimination (AUROC 0.92–0.99) and 98.3% adjacent accuracy suggest that ordinal regression heads, which penalize distant misclassifications more heavily than near misses, suit this task. Ordinal formulations similarly reduce grading-error magnitude in other medical imaging domains [29,30]. The confusion matrix’s concentration of errors at the mild-to-moderate boundary mirrors the intermediate-grade ambiguity that limits human inter-reader agreement [19,31]. Inter-reader agreement for foraminal stenosis is itself limited (weighted kappa 0.58 in the SPORT reliability substudy; Fleiss’ kappa 0.295 in a recent 12-reader study of the Lee classification), so the model may approach the ceiling imposed by label noise in the reference standard [19,31]. All grades come from a single expert reader, and model-reader agreement is bounded by that reader’s self-consistency (kappa 0.80–1.0 for the Lee system [15]); a QWK of 0.813 therefore likely sits near this noise ceiling, and materially exceeding it would require multi-reader consensus labels rather than architectural change. Against this literature, this study’s contribution is preserving the full four-grade ordinal scale rather than dichotomizing severity [24,27] and quantifying, under patient-clustered inference, how much interpretable morphometry adds to a matched image-only model.

The null contribution of morphometric features deserves careful interpretation. The anatomy-only branch reached QWK 0.444 against 0.314 for level and side indicators alone, leaving little agreement attributable to millimeter-scale measurements. The level-adjustment analysis showed the pooled spondylolisthesis and angulation correlations vanishing within level; these are not measurement failures but the anatomic reality that both foraminal geometry and disease prevalence vary systematically down the lordotic spine. Disc height, conversely, emerged as a genuine within-level correlate, matching the surgical expectation that disc collapse narrows the foramen. This dissociation carries a methodological caution: morphometric predictors reported without level adjustment may overestimate features that are simply proxies for spinal level, a trap prior quantitative work avoided by correlating within each lumbar level [32].

That image features alone matched fusion has practical implications: an appearance-only pipeline eliminates polygon segmentation at inference time, requiring only a cropped foraminal ROI from bounding-box annotations or automated detection. This is consistent with other high-performing lumbar stenosis classifiers that rely on CNN-based image classification without explicit morphometric extraction [24,25]. The two branches were also not fully independent sources of signal: each crop contains vertebral, discal, and, caudally, sacral context from which a CNN can implicitly infer level and side, so the appearance branch likely already encodes much of the level information the anatomy branch carries explicitly, further explaining the null fusion result. The morphometric features still aid interpretability and clinical communication: millimeter-scale disc height loss gives surgeons anatomically grounded context that a probability score does not. Deployment in a real clinical setting would additionally require automated foraminal localization across the full sagittal stack, for which detection stages exist in end-to-end systems [24,25]; integration with radiology reporting; probability recalibration; and prospective reader studies testing whether model output changes grading behavior. This distance between development and bedside adoption, not discrimination, is the binding constraint across spine artificial intelligence [33,34]. Interpretable bedside risk tools in orthopedics illustrate a viable translational path [35].

Several limitations warrant discussion. First, all labels derive from a single expert grading, so the reference standard itself contains noise that constrains achievable agreement [19,31]. Second, the morphometric descriptors come from a single mid-sagittal slice and cannot capture three-dimensional foraminal anatomy, including lateral disc herniations or facet hypertrophy visible only on parasagittal cuts. Perineural fat volume, a three-dimensional metric, correlates with stenosis grade across all lumbar levels, suggesting volumetric features could outperform the planar measurements used here [32]. Third, the dataset lacks clinical outcomes; whether grading correlates with symptoms, surgical candidacy, or postoperative improvement is unknown. Because stenosis severity is linked to surgical probability and the functional outcome [7,8], prospective validation against clinical endpoints is essential. Fourth, the comparison with the previously published benchmark of approximately 65% accuracy is indicative rather than head-to-head, as that work used a different data partition and the dataset provides no official split. Fifth, all imaging derives from one public dataset; the model has not been externally validated, nor evaluated in a complete clinical workflow in which foramina must first be localized across the full sagittal stack rather than supplied as pre-annotated ROIs. The reported performance therefore cannot be assumed to be representative of other institutions, scanners, acquisition protocols, or patient populations, and multi-center external validation is a precondition for clinical implementation. Finally, class imbalance limits statistical power for the moderate and severe grades, reflected in the wide confidence intervals for moderate-grade sensitivity (0.321, 95% CI 0.203–0.444). With only 53 moderate and 45 severe test foramina, grade-specific estimates should be interpreted cautiously and not generalized to cohorts with different grade distributions.

## 5. Conclusions

A fine-tuned convolutional neural network with an ordinal regression head achieved strong agreement for four-grade lumbar foraminal stenosis classification on sagittal T2-weighted MRI, with boundary discrimination that held across scanner vendors. The evaluated segmentation-derived morphometric features did not improve performance beyond imaging alone, and several apparent anatomic associations reflected confounding by lumbar level. Deep image features therefore appear to capture the relevant pathoanatomy more efficiently than hand-crafted geometric descriptors. External validation and evaluation within a complete clinical workflow are needed before implementation; further effort may be better spent on multi-rater consensus labels and clinical outcome validation than on more complex feature engineering.

## Figures and Tables

**Table 1:** Grading performance by model on the held-out test set.

Model	QWK (95% CI)	Accuracy	Balanced Acc.	Macro F1	Adjacent Acc.
Nominal softmax	0.806 (0.757–0.846)	0.766	0.632	0.652	0.974
Appearance-only	0.806 (0.756–0.844)	0.753	0.621	0.645	0.981
Anatomy-only	0.444 (0.359–0.518)	0.574	0.386	0.344	0.864
Fusion	0.813 (0.769–0.847)	0.753	0.603	0.628	0.983
Level + side only (reference)	0.314 (0.266–0.360)	0.521	0.344	0.270	0.909

Quadratic weighted kappa (QWK) with patient-clustered bootstrap 95% confidence intervals; accuracy is four-class. Adjacent accuracy counts predictions within one grade. *n* = 580 foramina (94 patients).

**Table 2:** (**a**). Per-grade one-vs.-rest sensitivity and specificity for the fusion model on the held-out test set. (**b**). Discrimination at each ordinal severity threshold for the fusion model on the held-out test set.

(**a**)			
**Grade**	***n***	**Sensitivity (95% CI)**	**Specificity (95% CI)**
Normal	378	0.878 (0.844–0.912)	0.787 (0.723–0.848)
Mild	104	0.567 (0.468–0.667)	0.836 (0.801–0.870)
Moderate	53	0.321 (0.203–0.444)	0.962 (0.943–0.979)
Severe	45	0.644 (0.510–0.774)	0.996 (0.991–1.000)
(**b**)			
**Ordinal Boundary**	***n* Positive**		**AUROC (95% CI)**
Normal vs. ≥ Mild	202		0.917 (0.895–0.939)
≤ Mild vs. ≥ Moderate	98		0.953 (0.938–0.968)
≤ Moderate vs. Severe	45		0.987 (0.976–0.995)

## Data Availability

The data use in this study are openly available in LSS-MRI-AISSLab dataset, is publicly available at https://data.mendeley.com/datasets/rgb77xm3jf/4 (accessed on 12 August 2026). All analysis code for this study, covering data preparation, morphometric feature extraction, model training, and evaluation, together with the exact patient-level train/test split indices (data/splits.csv), is openly available at https://github.com/rohanpa27/LSS_foraminal_stenosis_imaging (accessed on 12 August 2026) under an MIT license.

## Supplementary Materials

The following supporting information can be downloaded at https://www.mdpi.com/article/10.3390/jimaging12080388/s1, Figure S1: Association of each segmentation-derived morphometric feature with foraminal-stenosis grade, shown pooled (grey) and adjusted for lumbar level (orange), with patient-clustered bootstrap 95% confidence intervals. Level adjustment abolishes the apparent spondylolisthesis, angulation, and Posterior A associations and reveals a within-level disc-height association that pooling had masked.; Table S1: Morphometric feature associations with stenosis grade; Table S2: Performance by lumbar level and scanner vendor (fusion, test).

## References

[1] Katz J.N., Zimmerman Z.E., Mass H., Makhni M.C. (2022). Diagnosis and management of lumbar spinal stenosis. JAMA.

[2] Weinstein J.N., Tosteson T.D., Lurie J.D., Tosteson A., Blood E., Herkowitz H., Cammisa F., Albert T., Boden S.D., Hilibrand A. (2010). Surgical versus nonoperative treatment for lumbar spinal stenosis: Four-year results of the Spine Patient Outcomes Research Trial. Spine.

[3] Lurie J.D., Tosteson T.D.S., Tosteson A.S., Abdu W.A., Zhao W., Morgan T.S.M., Weinstein J.N.D. (2015). Long-term outcomes of lumbar spinal stenosis: Eight-year results of the Spine Patient Outcomes Research Trial (SPORT). Spine.

[4] Atlas S.J., Keller R.B., Robson D., Deyo R.A., Singer D.E. (2000). Surgical and nonsurgical management of lumbar spinal stenosis: Four-year outcomes from the Maine Lumbar Spine Study. Spine.

[5] Atlas S.J., Keller R.B., Wu Y.A., Deyo R.A., Singer D.E. (2005). Long-term outcomes of surgical and nonsurgical management of lumbar spinal stenosis: 8 to 10 year results from the Maine Lumbar Spine Study. Spine.

[6] Johnsson K.E., Rosén I., Udén A. (1992). The natural course of lumbar spinal stenosis. Clin. Orthop. Relat. Res..

[7] Kang D.H., Lee S., Kim H.J., Park S.M., Yeom J.S. (2022). Probability for surgical treatment in patients with lumbar spinal stenosis according to the stenotic lesion severity: A 5-10-year follow-up study. BMC Musculoskelet. Disord..

[8] Aaen J., Banitalebi H., Austevoll I.M., Hellum C., Storheim K., Myklebust T.Å., Anvar M., Weber C., Solberg T., Grundnes O. (2023). Is the presence of foraminal stenosis associated with outcome in lumbar spinal stenosis patients treated with posterior microsurgical decompression. Acta Neurochir..

[9] Boden S.D., Davis D.O., Dina T.S., Patronas N.J., Wiesel S.W. (1990). Abnormal magnetic-resonance scans of the lumbar spine in asymptomatic subjects: A prospective investigation. J. Bone Jt. Surg. Am..

[10] Amundsen T., Weber H., Lilleas F., Nordal H.J., Abdelnoor M., Magnaes B. (1995). Lumbar spinal stenosis: Clinical and radiologic features. Spine.

[11] Sirvanci M., Bhatia M., Ganiyusufoglu K.A., Duran C., Tezer M., Ozturk C., Aydogan M., Hamzaoglu A. (2008). Degenerative lumbar spinal stenosis: Correlation with Oswestry Disability Index and MR imaging. Eur. Spine J..

[12] Srinivas R., Phadke R., Salman S., Hazem D., Kaur H., Kumar R., Vaja S., Lee N.J. (2026). Endoscopic versus open lumbar decompression: A retrospective cohort study of 31,000 patients with 90-day follow-up. Neurosurg. Rev..

[13] Phadke R., Salman S., Kumar R., Paidisetty V., Matthews B., Srinivas R., Vaja S., Lee N.J. (2026). Endoscopic and percutaneous minimally invasive repair of pars interarticularis defects: A systematic review of clinical outcomes. Spine Deform..

[14] Salman S.G., Phadke R., Kumar R., Gill K., Vaja S., Lee N.J., Bono C. (2026). Long-term outcomes of lumbar total disc arthroplasty and hybrid constructs: A systematic review. Spine J..

[15] Lee S., Lee J.W., Yeom J.S., Kim K.-J., Kim H.-J., Chung S.K., Kang H.S. (2010). A practical MRI grading system for lumbar foraminal stenosis. AJR Am. J. Roentgenol..

[16] Schizas C., Theumann N., Burn A., Tansey R., Wardlaw D., Smith F.W., Kulik G. (2010). Qualitative grading of severity of lumbar spinal stenosis based on the morphology of the dural sac on magnetic resonance images. Spine.

[17] Lee G.Y., Lee J.W., Choi H.S., Oh K.J., Kang H.S. (2011). A new grading system of lumbar central canal stenosis on MRI: An easy and reliable method. Skelet. Radiol..

[18] Ko Y.-J., Lee E., Lee J.W., Park C.Y., Cho J., Kang Y., Ahn J.M. (2020). Clinical validity of two different grading systems for lumbar central canal stenosis: Schizas and Lee classification systems. PLoS ONE.

[19] Sá Silva J., Pereira A., Abreu V., Filipe J.P. (2025). Inter-observer variability in the classification of lumbar foraminal stenosis in magnetic resonance imaging using different evaluation scales. Eur. Spine J..

[20] Mamisch N., Brumann M., Hodler J., Held U., Brunner F., Steurer J. (2012). Radiologic criteria for the diagnosis of spinal stenosis: Results of a Delphi survey. Radiology.

[21] Andreisek G., Imhof M., Wertli M., Winklhofer S., Pfirrmann C.W.A., Hodler J., Steurer J., for the Lumbar Spinal Stenosis Outcome Study Working Group Zurich (2013). A systematic review of semiquantitative and qualitative radiologic criteria for the diagnosis of lumbar spinal stenosis. AJR Am. J. Roentgenol..

[22] Walsh P.J., Lee U.J., Lipetz J.S., Walz D.M. (2026). Improving reliability of MRI lumbar spinal stenosis assessment across radiology and spine specialties: Impact of a structured education intervention. Acad. Radiol..

[23] Yang X., Zhang Y., Li Y., Wu Z. (2025). Performance of artificial intelligence in diagnosing lumbar spinal stenosis: A systematic review and meta-analysis. Spine.

[24] Hallinan J.T.P.D., Zhu L., Yang K., Makmur A., Algazwi D.A.R., Thian Y.L., Lau S., Choo Y.S., Eide S.E., Yap Q.V. (2021). Deep learning model for automated detection and classification of central canal, lateral recess, and neural foraminal stenosis at lumbar spine MRI. Radiology.

[25] Tumko V., Kim J., Uspenskaia N., Honig S., Abel F., Lebl D.R., Hotalen I., Kolisnyk S., Kochnev M., Rusakov A. (2024). A neural network model for detection and classification of lumbar spinal stenosis on MRI. Eur. Spine J..

[26] Phadke R.A., Salman S.G., Salman Z.G., Yedupati S.M., Ong J., Tavakkoli A., Galhotra S., Tripuraneni A., Rizkalla J. (2026). Deep learning-based multi-class pediatric wrist fracture subtype classification: A pilot study comparing convolutional neural network architectures. J. Imaging.

[27] Lee Y.J., Liu C., Ting Y.H., Makmur A., Sng W.J., Cheng A.J.L., Tan J.H., Teo A.Q.A., Li Z., Ng A.H.Z. (2026). Deep learning models for lumbar spinal stenosis on MRI: Model comparison and clinical benchmarking. Eur. Spine J..

[28] Kumar R., Kaur H., Sporn K., Salman S.G., Phadke R., Sarnala S., Vaja S., Lee N.J., Lee N.J. (2026). Advancing spine connectomics and neural integration through machine learning and neuroengineering: A narrative review. Eur. Spine J..

[29] Toledo-Cortés S., Useche D.H., Müller H., González F.A. (2022). Grading diabetic retinopathy and prostate cancer diagnostic images with deep quantum ordinal regression. Comput. Biol. Med..

[30] Chen P., Gao L., Shi X., Allen K., Yang L. (2019). Fully automatic knee osteoarthritis severity grading using deep neural networks with a novel ordinal loss. Comput. Med. Imaging Graph..

[31] Lurie J.D., Tosteson A.N., Tosteson T.D., Carragee E., Carrino J., Kaiser J., Sequeiros R.T.B., Lecomte A.R., Grove M.R., Blood E.A. (2008). Reliability of readings of magnetic resonance imaging features of lumbar spinal stenosis. Spine.

[32] Gunasekaran V.S., Hejdak D., Meyer B., Klein A., Koch K. (2021). Quantitative correlation of lumbar foraminal stenosis with local morphological metrics. Eur. Spine J..

[33] Salman S.G., Phadke R., Kumar R., Zaman N., Tavakkoli A. (2026). Digital twins and multimodal artificial intelligence in spine care: A scoping review of concepts, evidence, and translational barriers. Spine Deform..

[34] Salman S., Phadke R., Kumar R., Momin A., Tavakkoli A. (2026). Risk prediction in spine surgery: A scoping review of traditional models, artificial intelligence, and the challenge of clinical translation. Spine Deform..

[35] Salman S.G., Phadke R., Carlin T., Rana A., Dawson J.R., Fitzgerald C.A., Seger C.P., Zielinski M.D., Dumas R.P. (2026). The fracture orthopedic risk of non-home discharge (FORD) score: A novel bedside predictive tool for non-home discharge in orthopedic trauma patients. Injury.

