# Supplementary material for: Ordinal Deep Learning for Lumbar Foraminal Stenosis Grading on Sagittal MRI
Source: J Imaging. 2026 Aug 19;12(8):388. doi: 10.3390/jimaging12080388 (PMC13514485; doi:10.3390/jimaging12080388)
Supplement: Supplementary file 1 [file jimaging-12-00388-s001.zip › jimaging-4499126-supplementary.pdf]

Journal of *Imaging*

MDPI

Supplemental materials

Ordinal Deep Learning for Lumbar Foraminal Stenosis Grading on Sagittal MRI

Rohan A. Phadke [1,*,†], Samer G. Salman [1,†], Zane G. Salman [2], Akhil Marupudi [3], Kirtan Patel [4], Joshua Ong [5], Alireza Tavakkoli [6], Sainyam Galhotra [7], Ajay Tripuraneni [1], James Rizkalla [8] and Nathan J. Lee [9]

[1] School of Medicine, Baylor College of Medicine, Houston, TX 77030, USA
[2] College of Natural Sciences, the University of Texas at Austin, Austin, TX 78712, USA
[3] McGovern Medical School, the University of Texas Health Science Center, Houston, TX 77030, USA
[4] Edward Via College of Osteopathic Medicine–Louisiana, Monroe, LA 71203, USA
[5] Massachusetts Eye and Ear, Harvard Medical School, Boston, MA 02114, USA
[6] Department of Computer Science, University of Nevada, Reno, NV 89557, USA
[7] Department of Computer Science, Cornell University, Ithaca, NY 14853, USA
[8] Department of Orthopaedic Surgery, Baylor University Medical Center, Dallas, TX 75246, USA
[9] Midwest Orthopaedics at Rush, Rush University School of Medicine, Chicago, IL 60612, USA
* Correspondence: rohan.phadke@bcm.edu
† These authors contributed equally to this work.

Table S1. Morphometric feature associations with stenosis grade

Feature	Normal	Mild	Moderate	Severe	Pooled rho (95% CI)	Level-adjusted rho (95% CI)
Disc height (mm)	11.179	11.333	11.18	11.139	0.021 (−0.030 to 0.073)	−0.108 (−0.163 to −0.054)
Disc-to-vertebral-body ratio	0.534	0.539	0.534	0.544	0.026 (−0.022 to 0.077)	−0.049 (−0.103 to 0.007)
Upper vertebral body height (mm)	20.802	21.218	21.274	20.808	0.074 (0.027 to 0.119)	−0.103 (−0.154 to −0.048)
Lower vertebral body height (mm)	21.269	20.942	20.68	20.332	−0.129 (−0.178 to −0.082)	−0.102 (−0.157 to −0.047)
Spondylolisthesis offset (mm)	3.149	−0.488	−1.576	−1.841	−0.349 (−0.387 to −0.310)	−0.000 (−0.055 to 0.053)
Spondylolisthesis ratio	0.078	−0.013	−0.041	−0.043	−0.355 (−0.393 to −0.315)	−0.001 (−0.055 to 0.052)
Segmental angulation (deg)	8.065	10.628	10.629	11.433	0.211 (0.171 to 0.252)	−0.036 (−0.081 to 0.009)
Disc anteroposterior width (mm)	41.204	43.164	44.536	45.321	0.296 (0.254 to 0.339)	0.195 (0.145 to 0.245)
Posterior A distance (mm)	76.005	79.842	79.596	81.095	0.146 (0.103 to 0.191)	−0.002 (−0.057 to 0.055)
Posterior B distance (mm)	140.58	141.294	140.5	142.006	0.024 (−0.024 to 0.071)	0.004 (−0.051 to 0.060)

 https://doi.org/10.3390/jimaging12080388

Table S1. Mean feature value by grade, with the Spearman correlation against ordinal grade reported pooled and adjusted for lumbar level. Lengths in millimeters unless a ratio or degree. Computed on all foramina, including the locked test set; these are descriptive only and no feature selection was performed on them. Denominators differ by feature: n = 2,935 foramina for disc height, disc-to-vertebral−body ratio, upper vertebral body height, disc anteroposterior width, and posterior distances, and n = 2,664 for lower vertebral body height, spondylolisthesis offset and ratio, and segmental angulation.

Table S2. Performance by lumbar level and scanner vendor (fusion, test)

Table S2. a. QWK by lumbar level. L1-L2 contains almost no higher-grade disease, making its kappa unstable.

Lumbar level	*n*	QWK fusion (95% CI)	QWK appearance−only (95% CI)
L1-L2	114	−0.013 (−0.039 to 0.000)	−0.024 (−0.052 to 0.000)
L2-L3	137	0.742 (0.561 to 0.855)	0.778 (0.574 to 0.893)
L3-L4	156	0.810 (0.714 to 0.875)	0.818 (0.731 to 0.881)
L4-L5	118	0.785 (0.707 to 0.846)	0.748 (0.631 to 0.830)
L5-S1	55	0.660 (0.366 to 0.802)	0.660 (0.403 to 0.792)

Table S2. b. Cross-vendor robustness of the fusion model.

Vendor	*n*	QWK fusion (95% CI)
GE	207	0.794 (0.681-0.869)
Philips	373	0.817 (0.762-0.856)

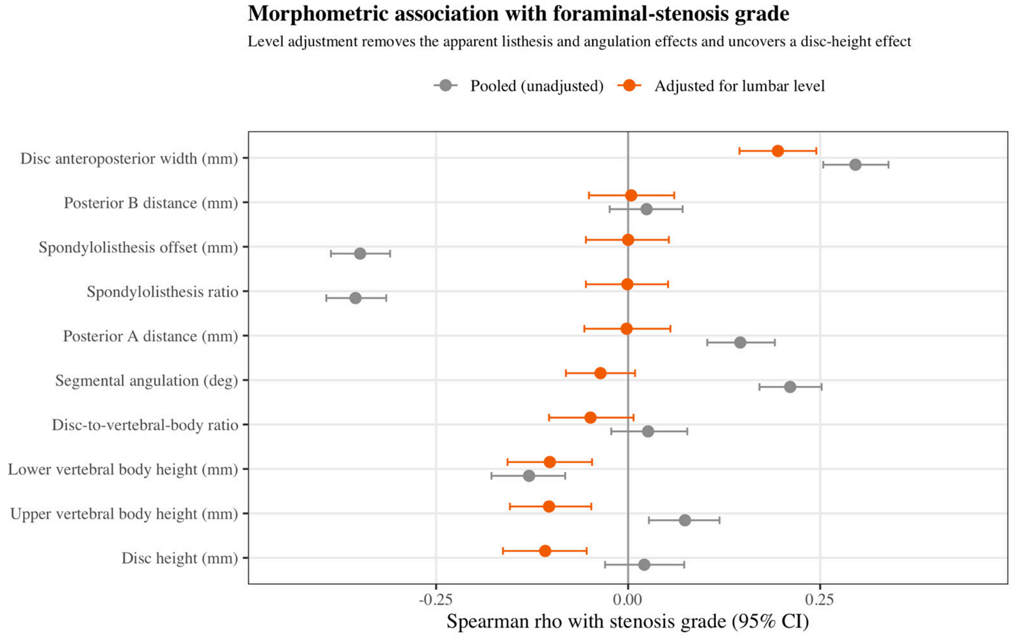

Figure S1. Association of each segmentation-derived morphometric feature with foraminal-stenosis grade, shown pooled (grey) and adjusted for lumbar level (orange), with patient-clustered bootstrap 95% confidence intervals. Level adjustment abolishes the apparent spondylolisthesis, angulation,

and Posterior A associations and reveals a within-level disc-height association that pooling had masked.
